# The Importance of the Deep Deltoid Ligament Repair in Treating Supination-External Rotation Stage IV Ankle Fracture: A Comparative Retrospective Cohort Study

**DOI:** 10.1155/2020/2043015

**Published:** 2020-11-29

**Authors:** Hongfeng Chen, Dongsong Yang, Zhen Li, Junke Niu, Pengru Wang, Qidi Li, Xishun He, Guangliang Wu

**Affiliations:** ^1^Department of Foot and Ankle Surgery, The Second Affiliated Hospital of Luohe Medical College, Western Haihe Road, Luohe, Henan 462300, China; ^2^Department of Orthopedic Surgery, The Second Affiliated Hospital of Luohe Medical College, Western Haihe Road, Luohe, Henan 462300, China

## Abstract

**Background:**

The necessity of the deep deltoid ligament repair in the treatment of supination-external rotation (SER) ankle stage IV fracture with deltoid ligament rupture is highly debated. We conducted this retrospective research aimed at exploring the curative effect of the deep deltoid ligament repair in treating SER fracture.

**Methods:**

Sixty-three patients with closed SER stage IV fractures received open reduction and internal fixation (ORIF), using either deep deltoid ligament repair (the DDLR group, 31 patients) or nondeep deltoid ligament repair (the NDDLR group, 32 patients). The radiographic parameters examined include the talocrural angle (TA), fibular length (FL), tibiomedial malleolar angle (TMMA), medial clear space (MCS), and tibiofibular clear space (TFCS). The functional performance parameters examined in the study were visual analog scale (VAS) pain score, American Orthopaedic Foot & Ankle Society (AOFAS) ankle-hindfoot scales, and range of motion of bilateral ankles (RMBA). Complications, including bone nonunion, infection, and fragment displacement, were also recorded and compared.

**Results:**

Similar basic characteristics were found in both cohorts. All patients completed follow-up ranging from 12 to 22 months (mean time: 12.41 ± 4.21 months). The DDLR group had significantly reduced VAS score (*p* < 0.05), with markedly increased RMBA (*p* < 0.05) compared to the NDDLR group. The two cohorts showed similar follow-up performance at 3 months (*p* > 0.05), 6 months (*p* > 0.05), and 12 months (*p* > 0.05), in terms of parameters including TA, FL, TMMA, MCS, TCS, and AOFAS ankle-hindfoot scales.

**Conclusion:**

Although similar radiographic performances were achieved in both cohorts, the DDLR group displayed enhanced functional outcome postsurgery, indicating that DDLR may be a better potential for the treatment of SER stage IV fracture with deltoid ligament rupture.

## 1. Introduction

Ankle fractures (AF) are one of the most common traumas involving the bone. Over the years, the incidence of AF increased rapidly, owing to the large elderly and athletic population [1, 2]. The severity of AF is also regulated by various comorbidities, including osteoporosis and diabetes [3, 4]. Supination-external rotation (SER) injury is a severe and frequent type of AF. It can affect the functional performance of the injured ankle [5]. In the presence of severe fracture, the decision to perform surgery depends on criteria like ankle displacement, size of the fracture fragment, stability of the ankle joint, and the degree of ligament injury [6].

It is well reported that, along with the trauma to the bone, SER injury can also bring about deltoid ligament damage due to a possible distal tibiofibular joint injury [7]. A prior arthroscopy study found the presence of deltoid ligament damage in 40% of patients with AF, and nearly 50% of AF patients had distal tibiofibular joint injury [8]. Furthermore, according to the Lauge-Hansen classification of AF, SER injury is the most prevalent type of AF with an incidence of 80% [9]. Given these odds, it may be fairly common to have a SER injury in combination with both deltoid ligament damage and distal tibiofibular joint injury. Although ORIF is widely accepted as a feasible and effective method of treatment for SER injury, there still exists a great controversy regarding the role of deep deltoid ligament repair in this process.

In this study, we sought to compare the radiographic and functional outcomes of surgical treatment of SER injury in patients with and without deep deltoid ligament repair. The authors hypothesized that patients with deep deltoid ligament repair would exhibit enhanced functional outcomes due to the rapid functional recovery of the ankle joint.

## 2. Methods

### 2.1. Patient Eligibility

Sixty-three patients with SER injury reported between March 2015 and March 2018 were included and analyzed retrospectively. Ethical approval and informed consent to participate in this study were retrieved from each patient. The following inclusion criteria were applied: (1) MCS≧5 mm and demonstrated as stage IV SER injury with distal tibiofibular damage, (2) patients aged ≧16 years old and with full possession of mental faculties, (3) able to tolerate the operation, and (4) intraoperative confirmation of deltoid ligament full-layer rupture and distal tibiofibular joint instability after reduction and fixation. The excluded criteria were as follows: (1) unable to tolerate surgery, (2) possessing open injury, (3) pathological fractures, and (4) chronic diseases and/or skeletal disorders.

### 2.2. Surgical Treatment and Rehabilitation Protocol

**Figure 1:**
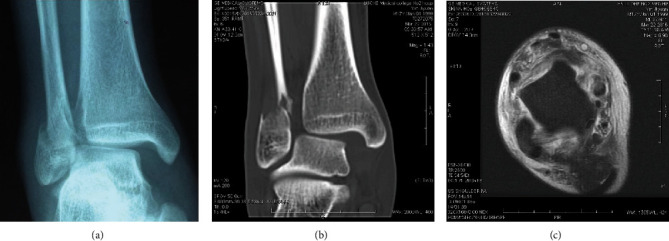
Preoperative imaging. (a) X-ray showed increased medial clear space (between the talus and medial malleolus). (b) CT demonstrated the distal tibiofibular joint separation with free fragments. (c) MRI showed full-thickness rupture of the deltoid ligament.

**Figure 2:**
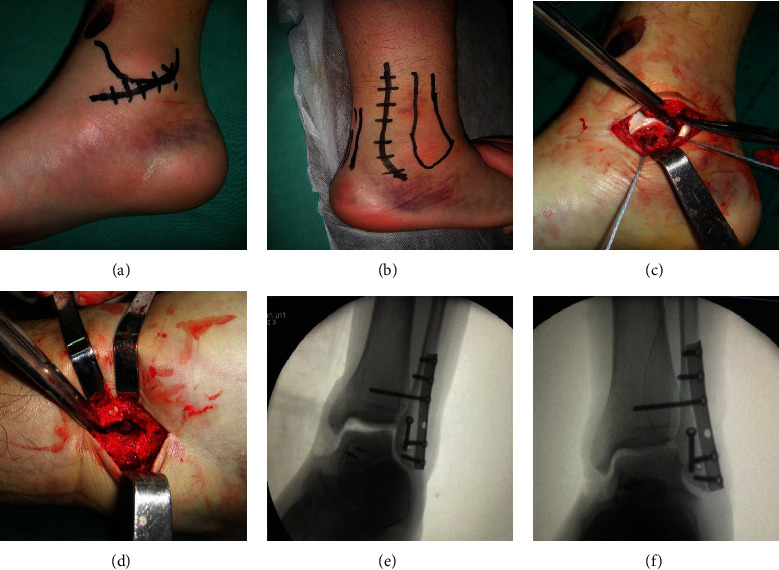
Intraoperative operations. (a, b) Medial and lateral incisions. (c) The anchor was placed in the talus at the talar insertion of the deep deltoid ligament. (d) Bone fragments in the distal tibiofibular joint were cleared, and the reduction was performed through a small anterior incision. (e) Intraoperative fluoroscopy showed an unsatisfactory medial mortise after fixation of the distal tibiofibular joint and repair of the deep deltoid ligament. (f) Intraoperative fluoroscopy revealed satisfactory medial mortise after the additional repair of the superficial deltoid ligament.

**Figure 3:**
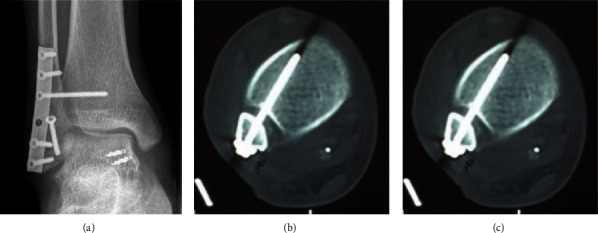
Postoperative imaging. (a) X-ray showed a commendable reduction and fixation. (b) CT demonstrated the position of the distal tibiofibular joint screw was good. (c) CT showed the reduction and alignment were satisfactory.

Preoperative imaging of a typical case in the DDLR group is shown in Figure 1. Patients were administrated spinal or general anesthesia, followed by placement in the supine position (Figure 2(a)). Then, the deltoid ligaments were exposed followed by the identification of the location of superficial and deep injuries of the deltoid ligaments. Next, fracture fragments were removed from the ankle cavity. To begin the correction process, two 3.5 mm suture anchors were placed in advance at the insertion point of the deep deltoid ligaments on the talus side without knotting. Anchor placing position was determined by the avulsed position on the talus where the remaining ligament tissue was found. Two anchors were placed anterior and posterior to the ligament endpoint on the talus (Figures 2(c) and 2(d)). The ligament was braided, not knotted. For ligament ruptures in the middle, two anchors were inserted at the ligament endpoint on the talus to suture and braid the ligaments. Then, the anchor suture was passed through the tunnel in the medial malleolus created by the K-wire. For the ligament rupture at the endpoint of the medial malleolus, two anchors were placed at the posterior colliculus and intercollicular groove without knotting. Subsequently, the patient was placed in a lateral position on the uninjured side. The posterior lateral approach was exposed through the space between the peroneus and flexor longus, and the lateral malleolus and posterior malleolus were fixed with a cortical bone screw (Figure 2(b)). On occasion of an intact posterior malleolus, the lateral malleolus was fixed with a cortical bone screw through the anterior space of the peroneus using the same incision. The patient was then placed back on a supine position. The distal tibiofibular joint was exposed through a small anterolateral incision to determine whether the distal tibiofibular joint was stable, free fracture fragments were cleared, and the distal tibiofibular joint was reduced under direct vision and fixed with a 3.5 mm cortical bone screw (Figures 2(e) and 2(f)). The medial anchor was knotted under moderate tension, and the superficial deltoid ligament was sutured with an absorbable suture. A femoral nerve block combined with an analgesia pump was given to relieve the postoperative pain. In the DDLR group, the ankle joint was fixed in a neutral position using an ankle brace for 2 weeks, while in the NDDLR group, the injured ankle was fixed using a varus plaster for four weeks. After the removal of the plaster or brace, the ankle joint was subjected to active and passive functional exercise without weight bearing. Around 8-12 weeks postsurgery, the distal tibiofibular screw was removed after careful X-ray examination, and the injured limb was allowed to begin weight-bearing rehabilitation exercises. Postoperative imaging is shown in Figure 3.

### 2.3. Assessment Index

Radiographic images were collected at each follow-up postsurgery. At twelve weeks postsurgery, radiographs of weight-bearing standing positions were imaged. At one-year postsurgery, the radiographs of the contralateral side were obtained for comparative analysis. The functional indices were assessed at the last follow-up, including TA, FL, TMMA, and MCS. Furthermore, the tibiofibular clear spaces (TFCS) for the syndesmoses were evaluated with weight-bearing radiographs and compared with the contralateral sides. In short, TA (talocrural angle) is the angle between a line drawn on the articular surface of the distal tibia and a line connecting the tips of the malleoli. A smaller TA suggests fibular shortening. TMMA is the angle formed between the anatomic axis of the tibia and the joint orientation line of medial malleolus. TMMA is used to assess the restoration of the alignment. MCS is the space between the articular surfaces of the talus and the medial malleolus. Greater than 4 mm width of MCS suggests a lateral shift of the talus. TFCS is a radiographic measure and is defined as the space between the groove of the distal tibial prominence and the medial margin of the distal fibula. It has been used in the diagnosis of syndesmotic injury and in the assessment of its repair. During the follow-up period, VAS pain score and the AOFAS ankle-hindfoot scale score were also measured. Joint range of motion (ROM) of the bilateral ankles was documented for comparative analysis.

### 2.4. Statistical Analysis

Statistical Product and Service Solutions (SPSS) software (version 15.0.1, SPSS Inc., Chicago, IL) was used to perform statistical analysis. The Wilcoxon rank-sum test was performed for the nonparametric data analysis, and *p* < 0.05 was assumed to be statistically significant.

## 3. Results

31 patients were treated with DDLR and 32 with NDDLR. Patients from the two groups had similar characteristics (Table 1), including average age (53.71 ± 7.42 and 52.89 ± 8.92 years, *p* = 0.3631), gender distribution (male : female (*n*), 17 : 14 and 15 : 17, *p* = 0.0826), body mass index (BMI (kg/m^2^), 27.13 ± 3.21 and 26.05 ± 5.14, *p* = 0.0721), and average VAS (7.42 ± 1.41 and 7.39 ± 1.67, *p* = 07.39 ± 1.67).

**Figure 4:**
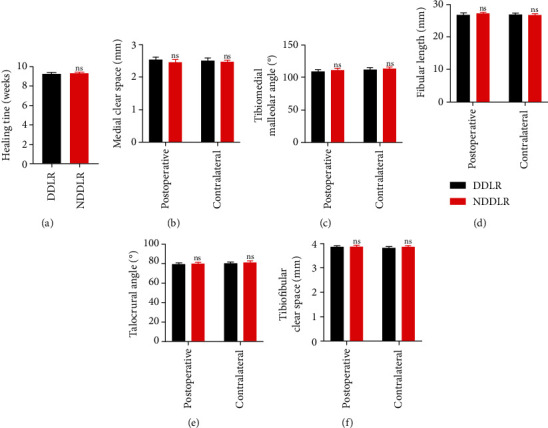
The radiographical results between the two cohorts. ^∗^*p* < 0.05, ^∗∗^*p* < 0.005.

The fracture healing time of the DDLR group was an average of 9.12 + 1.27 weeks, and the NDDLR group an average of 9.23 + 2.05 weeks. No significant difference was found in this index (*p* > 0.05). Similarly, there was no significant difference in the radiographic index, including MCS, TMMA, FL, TA, and TFCS, between the postoperative follow-up and the contralateral ankles in both the DDLR and NDDLR cohorts (Table 2, Figure 4).

**Figure 5:**
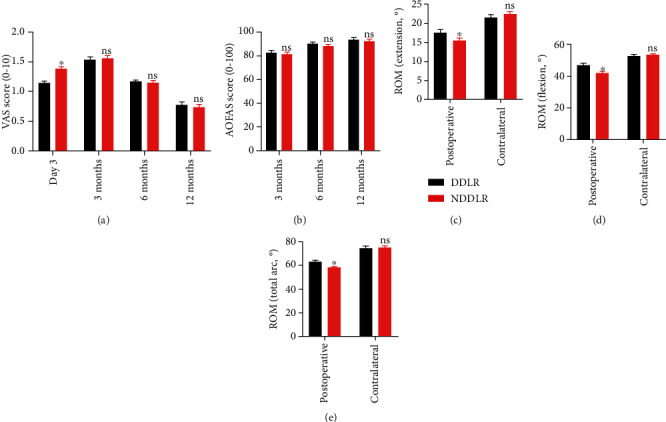
The functional results between the two cohorts. ^∗^*p* < 0.05, ^∗∗^*p* < 0.005.

The results of the functional evaluations in both groups are shown in Table 3 and Figure 5. On day 3 postsurgery, a significantly lower VAS score was found in the DDLR group compared to the NDDLR group (1.18 ± 1.07 and 1.36 ± 1.25, *p* = 0.0331). AOFAS ankle-hindfoot scale scores at follow-up found no significant differences between the two cohorts even though significant differences existed in the extension, flexion, and the total arc of ROM between both groups.

**Figure 6:**
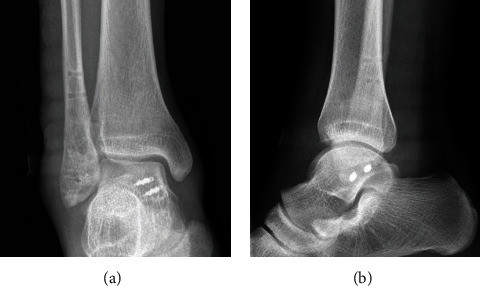
The imaging after internal fixation removal.

In total, 2 complications (6.5%) were observed in the DDLR group, of which 1 patient had lateral hardware irritation, and another one had a superficial infection in the incision area. In the NDDLR group, 2 complications (6.25%) were found, namely, 1 superficial infection and 1 superficial peroneal nerve paresthesia. The imaging after internal fixation removal of a typical case in the DDLR group is shown in Figure 6.

## 4. Discussion

In this study, the DDLR technique obtained satisfactory equal radiographic outcomes with far better functional performance as compared to the NDDLR method for stage IV SER ankle fractures. This study supported our hypothesis that patients with deep deltoid ligament repair would show better functional outcomes due to faster functional recovery of the ankle joint.

It is determined that the external twisting action that results in the SER injury, in turn, damages the anterior malleolus, lateral malleolus, posterior malleolus, medial malleolus, and the deltoid ligament to produce a range of fractures from weak to strong and categorized into stages I-IV according to the Lauge-Hansen classification of ankle fractures [10]. A considerable part of the stage IV SER injury not only damages the anterior distal tibiofibular ligament but also the interosseous ligament, leading to the instability of the distal tibiofibular joint. It is assumed that the deltoid ligament injury is present in one-quarter of the stage IV SER injury [11]. There is great controversy regarding the importance of the repair of the deltoid ligament in treating injury to the distal tibiofibular ligament. Currently, more reports have surfaced suggesting a major role of the deltoid ligament repair in improving clinical outcomes of the distal tibiofibular ligament [12, 13]. For instance, one report suggested that suture anchors could effectively maintain the stability of ankle mortise after anatomic steel plate or screw internal fixation of AF and, thereby, effectively repair the torn deltoid ligament [14]. Similarly, another study revealed that repair of the deltoid ligament could significantly reduce the postoperative MCS and the rate of defective reduction [15]. We assumed that if the MCS remains normal after the fixation of the lateral malleolus and posterior malleolus then the distal tibiofibular joint is not needed to fix. In this retrospective study, 63 patients with stage IV SER injury were analyzed. In one case, C-arm fluoroscopy indicated that the MCS was widened after the fixation of the lateral malleolus and the distal tibiofibular joint. Furthermore, the MCS did not improve after suturing the deep layer of the deltoid ligament. Therefore, it was assumed that the repair of the deep layer of the deltoid ligament alone could not provide enough strength to alleviate the MCS. As a result, we suggest both layers of the deltoid ligament be repaired as an effective treatment for stage IV SER injury patients.

The deep deltoid ligament is not easy to expose. Therefore, the NDDLR method offered a traditional and feasible approach to reverse the MCS in stage IV SER injury patients. A recent study indicated that the fixation of the foot at the varus position could provide an auxiliary effect to AF patients with deltoid ligament injury after ORIF [16]. However, the curative effect of NDDLR in stage IV SER injury patients remains elusive. In the present study, we compared the DDLR and the NDDLR technique in treating stage IV SER injury. We revealed similar radiographic results and equivalent AOFAS ankle-hindfoot scale scores in both cohorts. However, the DDLR group exhibited lower early pain scores, a shorter postoperative stay, and a better ROM indicating that the DDLR could be a potentially better alternative for the treatment of stage IV SER injury.

Similarly to other studies, the present study had limitations. Among them are the retrospective nature in which the two cohorts underwent surgical intervention, the limited number of subjects, and the absence of a long-term study. Additionally, the DDLR technique is surgeon dependent and requires a learning curve. With expertise and shorter operation duration, the DDLR approach would prove to be more effective. Our recommendation is to consider DDLR in treating AF, especially if the surgeon is proficient in ORIF in treating AP. This will not only shorten the learning curve but also prevent further complications.

## 5. Conclusion

Taken together, it is concluded that both DDLR and NDDLR approaches are safe, reliable, and effective in the treatment of stage IV SER injury. DDLR achieves satisfactory performance with less postoperative pain, faster recovery, and better ROM as compared to NDDLR, indicating that DDLR a potentially better option for treating stage IV SER fractures.

## Figures and Tables

**Table 1:** The characteristics of the two cohorts.

	DDLR (*n* = 31)	NDDLR (*n* = 32)	*p* value
Age (year, mean ± SD)	53.71 ± 7.42	52.89 ± 8.92	0.3631
Sex (male : female, *n*)	17 : 14	15 : 17	0.0826
BMI (kg/m^2^, mean ± SD)	27.13 ± 3.21	26.05 ± 5.14	0.0721
VAS pain score (0-10), mean	7.42 ± 1.41	7.39 ± 1.67	0.7356

BMI: body mass index.

**Table 2:** The radiographic results between the two cohorts.

	DDLR group	NDDLR group	*p* value
Healing time	9.12 ± 1.27	9.23 ± 2.05	0.8167
MCS (mm)			
Postoperative	2.52 ± 0.47	2.48 ± 0.47	0.6547
Contralateral	2.49 ± 0.52	2.47 ± 0.51	0.5598
TMMA (°)			
Postoperative	109.42 ± 6.27	111.07 ± 5.83	0.3986
Contralateral	110.12 ± 5.79	112.27 ± 5.92	0.1375
FL (mm)			
Postoperative	27.12 ± 2.25	27.35 ± 2.76	0.7426
Contralateral	26.92 ± 2.43	26.44 ± 2.09	0.9463
TA (°)			
Postoperative	78.11 ± 3.21	79.41 ± 2.98	0.2436
Contralateral	79.12 ± 2.94	79.42 ± 3.39	0.8214
TFCS (mm)			
Postoperative	3.82 ± 1.31	3.87 ± 1.57	0.1534
Contralateral	3.79 ± 1.27	3.84 ± 1.29	0.0776

DDLR: deep deltoid ligament repair; NDDLR: nondeep deltoid ligament repair; MCS: medial clear space; TMMA: tibiomedial malleolar angle; FL: fibular length; TA: talocrural angle; TFCS: tibiofibular clear space.

**Table 3:** The functional results between the two cohorts.

	DDLR group	NDDLR group	*p* value
VAS score			
Day 3	1.18 ± 1.07	1.36 ± 1.25	0.0331
3 months	1.52 ± 1.21	1.54 ± 1.19	0.2453
6 months	1.17 ± 1.05	1.16 ± 1.12	0.7232
12 months	0.79 ± 0.71	0.78 ± 0.65	0.5783
AOFAS score			
3 months	81.41 ± 8.56	80.271 ± 7.75	0.2457
6 months	89.12 ± 9.17	87.22 ± 9.04	0.0968
12 months	92.81 ± 11.49	91.56 ± 10.86	0.5562
ROM (°)			
Extension			
Postoperative	17.10 ± 5.13	14.21 ± 5.54	0.0281
Contralateral	21.56 ± 6.08	22.01 ± 6.26	0.4687
Flexion			
Postoperative	46.86 ± 6.17	41.53 ± 6.64	0.0168
Contralateral	52.35 ± 7.09	52.62 ± 6.94	0.7562
Total arc			
Postoperative	62.52 ± 7.15	57.35 ± 6.90	0.0491
Contralateral	73.15 ± 8.19	74.51 ± 8.56	0.6342

DDLR: deep deltoid ligament repair; NDDLR: nondeep deltoid ligament repair; VAS: visual analog scale; AOFAS: American Orthopaedic Foot & Ankle Society; ROM: range of motion.

## Data Availability

All data generated during this study are included in this published article.
